# Simplified assessment of castration-induced pain in pigs using lower complexity algorithms

**DOI:** 10.1038/s41598-023-48551-1

**Published:** 2023-12-01

**Authors:** Gustavo Venâncio da Silva, Giovana Mancilla Pivato, Beatriz Granetti Peres, Stelio Pacca Loureiro Luna, Monique Danielle Pairis-Garcia, Pedro Henrique Esteves Trindade

**Affiliations:** 1 Laboratory of Applied Artificial Intelligence in Health (LAAIH), Department of Anesthesiology, Botucatu Medical School, São Paulo State University (Unesp), Botucatu, São Paulo Brazil; 2 Department of Veterinary Surgery and Animal Reproduction, School of Veterinary Medicine and Animal Science, São Paulo State University (Unesp), Botucatu, São Paulo Brazil; 3 Global Production Animal Welfare Laboratory, Department of Population Health and Pathobiology, College of Veterinary Medicine, North Carolina State University (NCSU), Raleigh, NC USA

**Keywords:** Animal behaviour, Diagnostic markers, Quality of life

## Abstract

Pigs are raised on a global scale for commercial or research purposes and often experience pain as a by product of management practices and procedures performed. Therefore, ensuring pain can be effectively identified and monitored in these settings is critical to ensure appropriate pig welfare. The Unesp-Botucatu Pig Composite Acute Pain Scale (UPAPS) was validated to diagnose pain in pre-weaned and weaned pigs using a combination of six behavioral items. To date, statistical weighting of supervised and unsupervised algorithms was not compared in ranking pain-altered behaviors in swine has not been performed. Therefore, the aim of this study was to verify if supervised and unsupervised algorithms with different levels of complexity can improve UPAPS pain diagnosis in pigs undergoing castration. The predictive capacity of the algorithms was evaluated by the area under the curve (AUC). Lower complexity algorithms containing fewer pain-altered behaviors had similar AUC (90.1–90.6) than algorithms containing five (89.18–91.24) and UPAPS (90.58). In conclusion, utilizing a short version of the UPAPS did not influence the predictive capacity of the scale, and therefore it may be easier to apply and be implemented consistently to monitor pain in commercial and experimental settings.

## Introduction

Pigs (*Sus scrofa domesticus*) are raised worldwide for commercial or research purposes^1,2^. During their lifetime, pigs are routinely submitted to painful procedures^3,4^, with castration commonly performed on most male pigs in commercial and research settings to improve meat quality and reduce the risk of injury associated with aggression^5,6^. Despite the immunocastration raising popularity in the global swine industry^7^, studies estimated that 61% of European male pigs^8^ and up to 94 million male piglets in the United States^9^ are surgically castrated annually. In a production context, painful conditions such as surgical castration can decrease performance and result in poor weight gain^10^, while in experimental frameworks, pain experienced by the animal can add bias to the scientific research results^11^. Regardless of either scenario, the pig’s welfare is compromised thus presenting an ethical and legal dilemma^11^ that needs to be addressed both on-farm and in the laboratory^4,12^.

Pain is defined as “an unpleasant sensory and emotional experience associated with actual or potential tissue damage, or described in terms of such damage”^13^. For humans, the gold-standard method for pain assessment is through self-reporting^14^, however, in non-verbal animals, such as swine, methods to assess pain vary and include deviations to the animal’s physiological (e.g. infrared thermography^15^, cortisol^16^ and prostaglandin-2^17^) and behavioral response to post-painful procedure (e.g. pain scales^18–21^, time budget^16,22–27^, frequency of pain-associated behavioral expression^16,24,27–29^). Behavioral pain assessment is considered more favorable given it is non-intrusive, non-invasive, cost-effective, and easier to assess across diverse farm or laboratory settings^17^. However, many veterinarians and farmers struggle with pain assessment in pigs^30,31^. In a previous study, 32.8% of farmers and 40.4% veterinarians agreed that “it is difficult to recognise pain in pigs”^3^, and in other, only 32% of canadian veterinarians considered to have an adequate knowledge of analgesia in pigs^31^. To help mitigate this challenge, pain scales such as the Unesp-Botucatu Pig Composite Acute Pain Scale (UPAPS) have been developed based on pain-altered behaviors^18^ and validated as means to assess pain states after surgical castration using recorded videos^18,19^. Currently, the UPAPS is composed of either five (pre-weaned pigs) or six (weaned pigs) behavioral items categorized further into four scores^18,19^.

Despite the advantages of the behavior-based pain scales in recognized animals pain, its use can be laborious particularly when the scale relies on several pain-altering behavioral items to be assessed simultaneously. Pain scales for humans and other species have benefited from statistical weightings and improvements suggested by supervised and unsupervised algorithms^32–35^ to identify what behavioral items are more responsive (altered) than others. Supervised algorithms require a response variable to adjust the algorithm to account for conditions, such as painful or pain-free, while unsupervised algorithms do not use a response variable^36^. These algorithms were used to rank behaviors of importance, which can result in not only improvements to the scale itself but may improve the veterinarians and farmers experience assessing pain when accomplished in a more efficient, less time-consuming and simple manner.

Recently, our research team has demonstrated the weighted importance of pain-altered facial expression in horses using principal component analysis (unsupervised algorithm)^35^, in sheep using binomial multilevel logistic regression and random forest^32^ and in swine using binomial multilevel logistic regression (supervised algorithms)^34^. To date, no studies have been conducted in swine comparing supervised and unsupervised algorithms for weighting of pain-altered behaviors across ages (pre-weaned and weaned) and no work has compared the accuracy of multiple algorithms with different levels of complexity and variables. Therefore, the aim of this study was to verify if supervised and unsupervised algorithms with different levels of complexity can improve UPAPS diagnosis in weaned and pre-weaned pigs undergoing castration. Our hypothesis was that lower complexity algorithms might improve UPAPS diagnosis.

## Results

### Binomial multiple logistic regression (LR)

**Figure 1:**
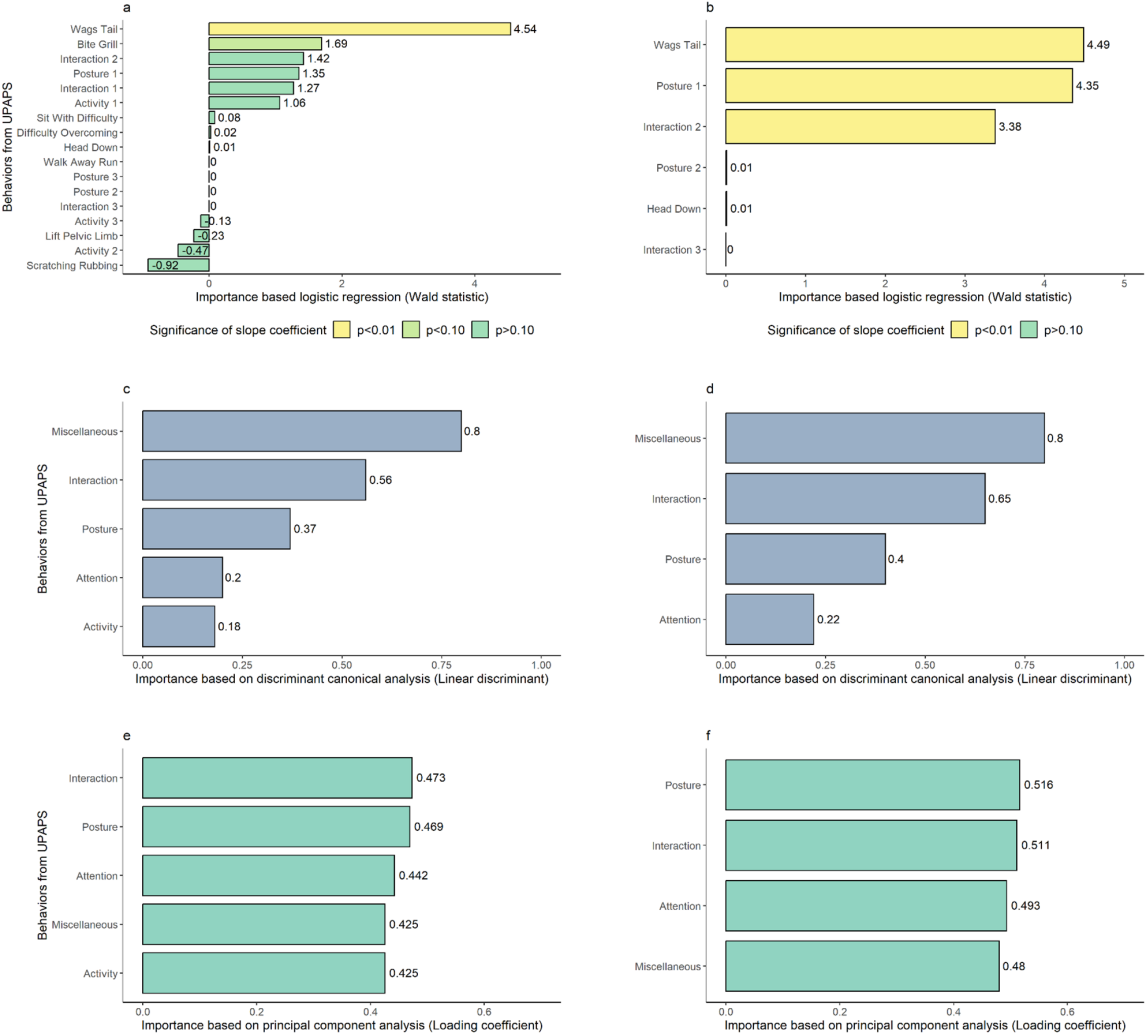
Importance of the pain-altered behaviors of the Unesp-Botucatu Pig Composite Pain Scale based on (**a**) Full logistic regression, (**b**) Refined logistic regression, (**c**) Full discriminant canonical analysis and (**d**) Refined discriminant canonical analysis, (**e**) Full principal component analysis, and (**f**) Refined principal component analysis.

Logistic Regression algorithms indicated the significance of each pain-altered behavior contribution to the pain-free or painful condition. From 17 pain-altered behaviors of the UPAPS, the Full LR only had Wags Tail (wags tail continuously and intensely) with a significant contribution (p < 0.001) to the algorithm (Table 1), which was also the most important pain-altered behavior according to the Wald statistics of Full LR (Fig. 1a). A Refined LR was then conducted to select the predictor variables for the best algorithm based on the best subsets technique using the Bayesian information criterion (BIC) as a ranking criterion. The BIC values were lower in Refined LR (72.0) than in Full LR (142.5), demonstrating a better adjustment of the algorithm after refinement. Six pain-altered behaviors were retained in the Refined LR. Wags Tail (wags tail continuously and intensely), Posture 1 (changes posture with some discomfort), and Interaction 2 (occasionally moves away from the other animals, but accepts approaches and shows little interest in the surroundings) contributed significantly (p < 0.001) to the Refined LR (Table 2), which were also the three most important pain-altered behaviors respectively (Fig. 1b). The three pain-altered behaviors related to activity were excluded in the Refined LR.

**Table 1:** Parameters of the full binomial multiple logistic regression algorithm.

Parameters	Estimate	Standard error	p-value
Linear coefficient (α)	− 3.928	0.711	< 0.001
Slope coefficients (β)			
Posture 1	3.189	2.369	0.178
Posture 2	22.926	4712.514	0.996
Posture 3	8.423	11,217.260	0.999
Interaction 1	1.498	1.178	0.203
Interaction 2	8.004	5.648	0.156
Interaction 3	12.918	13,083.750	0.999
Activity 1	1.218	1.144	0.286
Activity 2	− 2.614	5.541	0.637
Activity 3	− 0.633	4.939	0.897
Lift pelvic limb	− 0.995	4.282	0.816
Scratching rubbing	− 4.571	4.953	0.356
Walk away run	15.147	4682.143	0.997
Sit with difficulty	0.167	2.216	0.939
Wags tail	5.768	1.272	< 0.001
Bite grill	1.717	1.0187	0.092
Head down	21.683	4119.689	0.996
Difficulty overcoming	0.118	5.004	0.981

Pain-free (before castration) or painful (after castration) condition was used as a predictive variable and dummy of each pain-altered behavior of the Unesp-Botucatu Pig Composite Pain Scale as predictor variables.

**Table 2:** Parameters of the refined binomial multiple logistic regression algorithm.

Parameters	Estimate	Standard error	p-values
Linear coefficient (α)	− 3.093	0.457	< 0.001
Slope coefficients (β)			
Posture 1	5.045	1.161	< 0.001
Posture 2	20.221	3067.421	0.995
Interaction 2	4.194	1.241	< 0.001
Interaction 3	20.997	4684.459	0.996
Wags tail	5.180	1.153	< 0.001
Head down	20.688	2684.248	0.993

Pain-free (before castration) or painful (after castration) condition was used as a predictive variable and dummy of each pain-altered behavior of the Unesp-Botucatu Pig Composite Pain Scale as predictor variables.

### Discriminant canonical analysis (CDA)

Full CDA was performed using the five UPAPS items (Posture, Interaction, Activity, Attention and Miscellaneous) as grouping variables as pain-free or painful condition as a response variable. Refined CDA was performed using four UPAPS items, excluding the item Activity and using Condition as response variable. As Condition is a binomial variable, the algorithms generated only one canonical discriminant function, which accounted for 100% of variation in both cases. Miscellaneous item had the greater linear discriminant for both Full and Refined CDA (0.80 in both algorithms), while in Full CDA the smaller linear discriminant was from Activity item (0.18) and in Refined CDA it was from Attention item (0.22) (Fig. 1c and d).

### Principal component analysis (PCA)

Full PCA was performed using the five UPAPS items and five principal components (PC) were generated. Horn’s parallel analysis indicated only the retention of the first principal component (PC1). The PC1 accounted for 72.45% of variance and eigenvalue of 3.62. For variance and eigenvalue of all principal components please see Table S1. The Interaction item had the higher loading value (0.47), while the Activity item had the lower (0.42) (Fig. 1e). Refined PCA was performed using four UPAPS items, excluding the Activity item, generating four PCs. Horn’s parallel analysis also indicated only the retention of the PC1. In this algorithm, PC1 accounted for 76.16% of variance and eigenvalue of 3.04. For variance and eigenvalue of all principal components please see Table S2. The Posture item had the higher loading value (0.52), while the Miscellaneous item had the lower (0.48) (Fig. 1f).

### Predictive capacity

All areas under the curve (AUCs) from receiver operating characteristic (ROC) curves generated from the algorithms output were above 90%, except for Refined LR that was 89.18% (Table 3). No algorithm was statistically different from UPAPS by DeLong test (p > 0.05). Sensitivity estimates (median) ranged from 0.88 to 0.90, while specificity estimates (median) ranged from 0.86 to 0.90.

**Table 3:** Area under the curve (AUC) from receiver operating characteristic (ROC) curves of each algorithm and the Unesp-Botucatu Pig Composite Pain scale.

ROC curve	AUC (%)	p-value (UPAPS vs)	Threshold	Sensitivity	Specificity
UPAPS	90.58 (84.32–96.84)	NA	2.50 (1.50–3.50)	0.90 (0.8–0.98)	0.88 (0.78–0.98)
UPAPS without activity	91.24 (85.33–97.15)	0.317	2.00 (1.50–2.50)	0.90 (0.78–0.96)	0.88 (0.78–0.96)
Full LR	90.6 (84.57–96.63)	0.980	0.86 (0.15–0.88)	0.88 (0.78–0.98)	0.90 (0.76–0.98)
Refined LR	89.18 (82.72–95.64)	0.358	0.40 (0.40–0.99)	0.90 (0.76–0.98)	0.86 (0.74–0.94)
Full PCA	91.32 (85.29–97.35)	0.271	0.92 (0.45–1.32)	0.90 (0.80–0.98)	0.90 (0.80–0.98)
Refined PCA	91.52 (85.60–97.44)	0.129	1.01 (0.50–1.48)	0.90 (0.80–0.98)	0.90 (0.80–0.98)
Full CDA	90.56 (84.45–96.67)	0.982	0.86 (0.86–1.42)	0.90 (0.78–0.98)	0.88 (0.76–0.96)
Refined CDA	90.12 (83.80–96.44)	0.603	0.93 (0.93–1.45)	0.90 (0.78–0.98)	0.88 (0.76–0.96)

Data are presented as median (95% confidence interval). AUC was compared based on DeLong test.

*LR* binomial multiple logistic regression, *PCA* principal component analysis, *CDA* canonical discriminant analysis, *NA* not applied.

P-value refers to DeLong test, applied to compare AUC between the specified ROC curve and Unesp-Botucatu Pig Composite Pain Scale ROC curve.

## Discussion

Castration-induced pain is a critical welfare issue that can be a legal and ethical obligation for swine used for research and husbandry purposes and evaluating deviations to the pig’s behavioral response is an effective means to diagnosing pain accurately^17^. Unesp-Botucatu Pig Composite Acute Pain Scale (UPAPS) is a species-specific tool developed for assessing swine pain and has been validated for use in weaned^18^ and pre-weaned pigs^19^ undergoing castration. Because simultaneous assessment of multiple pain-altering behaviors may be difficult, we used statistical weightings to graduate the importance of behavioral items to facilitate using the scale. Therefore, we investigated if supervised and unsupervised algorithms with different levels of complexity improved UPAPS diagnosis across weaned and pre-weaned pigs.

This study utilized LR, CDA and PCA algorithms to assess the importance of pain-altered behaviors used in the UPAPS. The results from this study demonstrated that lowering algorithm complexity by removing the Activity item preserved the predictive capacity when applying the weightings using CDA and PCA. These techniques generated parameters that were applied to ranking pain-altered behaviors and all Refined algorithms had statistically similar AUC to Full algorithms with an AUC above 89%. Activity item comprises behavioral responses that are increased in painful conditions in some studies^17,28^ and decreased in another^37^. Additionally, younger pigs are less affected behaviorally by castration-induced pain than older ones^38^. Activity behaviors as described in UPAPS are known to rely on housing conditions, which depends on both the animal facility structure and/or guidelines and on animals age^39^. These three factors might explain why Activity pain-altered behaviors were consistently less important in some algorithms when two datasets including weaned and pre-weaned pigs were merged. Another explanation for the apparent less importance of the Activity item is overlapping with pain-altered behaviors in Posture and Interaction items, which might be caused by description similarities^18,23^. The Activity item was considered with satisfactory consistency, inter- and intra-observer reliability in previous studies^18,19^. In a recent study, Activity pain-altered behaviors had high statistical importance^34^. We reasoned that this might be caused by methodological differences. In previous studies of weighting UPAPS castration-induced pain-altered behaviors, the response variable was the observer analgesia indication^34^, while in ours it is the condition (painful or pain-free). Altogether, such pieces of evidence suggest the removal of the Activity item from UPAPS when applying the weightings by CDA or PCA across ages for diagnosing castration-induced pain. This specific finding also gave us the insight that the importance of each pain-altered behavior might not be closely related to consistency or observer reliability, and the relationship between them could be investigated in the future.

Posture and Interaction items were consistently important for all algorithms. In Refined LR, two out of three pain-altered behaviors with a significant slope coefficient were from Posture and Interaction items. Both CDA discriminant coefficients and PCA loading values also indicated Posture and Interaction items as one of the most important items of the UPAPS. Posture and Interaction items comprise castration-induced pain-altered behaviors that are similar to behaviors found to be altered in other studies^23,25,28,37,38^ thus supporting their importance. In a previous study, where UPAPS was weighted following a binomial multilevel logistic regression using a weaned pigs dataset, Posture and Interaction behaviors were also of high importance^34^.

In LR and CDA algorithms, Miscellaneous item (CDA) or its individual pain-altered behaviors (LR) were indicated as one of the most important, while in PCA, this item was one of the least important. This difference can be partially explained due to LR and CDA being supervised techniques, in other words, it uses a response variable, while PCA is an unsupervised technique, it does not need a response variable^36^. Since PCA loading values may be interpreted as the amount of variance that a variable had^40^, we might argue that Miscellaneous pain-altered behaviors varied less than the other ones, but when it occurred, it contributed significantly to the response variable outcome. Miscellaneous pain-altered behaviors are likely correlated with the response variable shift (painful and pain-free condition) and this can be partially explained because it is composed of behaviors related to castration-induced pain or discomfort, while part of the other UPAPS items are related to maintenance behaviors that can or cannot be altered when the pig is experiencing pain. These results reinforce the need for the comparison between techniques, as demonstrated on sheep^32^. In addition, because the majority of validation steps are unsupervised techniques, future refinement and validation processes may benefit from the use of LR and CDA, as suggested previously^41^.

Changes in UPAPS were expected since this is the first time a supervised algorithm was applied to weight the castration-induced pain-altered behaviors of the scale using a dataset of weaned and pre-weaned piglets. In Full LR algorithm, only the slope coefficient from the Wags Tail behavior was statistically significant, while there were other slope coefficients that had negative estimates. These results combined suggest a poor adjustment of the algorithm, which supported a refinement in which pain-altered behaviors should be considered. Refined LR had the lowest BIC combination of pain-altered behaviors and it was the best-adjusted algorithm. Also in Refined LR, we found three pain-altered behaviors with low Wald statistics and high standard error: Posture 2 (Changes posture, with discomfort, and protects the affected area), Head Down and Interaction 3 (Moves or runs away from other animals and does not allow approaches; disinterested in the surroundings). Considering that Refined LR was the best-adjusted algorithm, these three items might occur in agreement with the response variable.

This study is not free of limitations. First, all studies in pain-altered behavior must face the fact that in some species, the pain perception threshold is altered by negative affective states, such as anxiety and distress^4^. In agreement with that, there are no behaviors that exclusively address pain, but the assessment of pain-altered behaviors substantially contributes to identifying pain^42^. In our study, some dissimilarities in housing might affect the behavior response^43^. Also, UPAPS Appetite item was not considered because it had no statistical significance in our previous study^34^, however, altered feeding behavior was reported in another research as a pain indicator for pre-weaned piglets^44^. Another limitation of this study was the unbalanced number of pigs in each dataset. Although there was not a sign of underfitting according to AUC, sensitivity and specificity of the algorithms, further studies could increase the sample size. In addition, the difference in the pain control protocol between the databases due to the legislation for each host country represents a study limitation. Lastly, timing of observations was slightly different between the two datasets merged in the current study, however, they represent the same conditions.

Realistically, this study might improve the practice of veterinarians who consider their knowledge not sufficient for assessing pain in pigs^31^ or for farmers and veterinarians who find this evaluation difficult^30^, although this was not tested yet. The AUC from UPAPS original weighting without the Activity item was statistically similar to the full UPAPS, which supports a shorter version of the scale. A shorter version of UPAPS may be easier to apply with fewer items, increasing the chance for its employment and regular use in commercial and experimental contexts. Our study considered surgical castration-induced pain to refine the UPAPS, however, some UPAPS pain-altered behaviors are related to the surgical areas, and the scale also might be helpful for pain diagnosing due to surgeries performed in the same area of the pig body. Also, UPAPS maintenance behaviors might be a general contribution for pain recognition from other sources. These two points may be relevant since the UPAPS pain-altered behaviors are easily recognizable by evaluators in the tutorial videos on the Animal Pain webpage (https://animalpain.org/en/home-en/). Both extrapolations of our findings require to be tested by clinical studies assessing multiple painful conditions, however, it is very likely that in other contexts, with different types, areas, durations and or intensities of pain, the UPAPS would need further adaptations that could employ the same rationale used in the present study.

Behavioral methods for assessing pain such as UPAPS are essential in recognizing and quantifying pain in animals^45^ and therefore their shortening and usability refinement contributes not only to improving pig pain diagnosis but also to welfare. The average time to score original and shortened UPAPS as well as the potential gain of accuracy of the shortened UPAPS should be assessed in future studies. Yet, the shortened scale might be used for developing software that automates pain diagnosis. The present study also reinforces the importance of employing supervised and unsupervised algorithms to rank pain-altered behaviors.

We concluded that lowering the complexity of supervised and unsupervised algorithms for the statistical weighting of UPAPS is beneficial and helped to identify important behaviors and suggest a potential more efficient acute pain scale to be used in piglets undergoing surgical castration with no impairment in predictive capacity. Further studies might confirm or not our findings by monitoring piglets pain in a real-world setting.

## Methods

In the current study data was obtained from two previous publications^18,19^. The first study was approved by the Ethical Committee for the Use of Animals in Research of the School of Veterinary Medicine and Animal Science, Unesp, Botucatu, Brazil, under protocol number 102/2014 and followed the Brazilian Federal legislation of National Council for the Control of Animal Experimentation (CONCEA)^18^. The second study was approved by the North Carolina State University Animal Care and Use Committee under protocol number 19-796^19^. Both previous publications and the current study followed ARRIVE guidelines for animal research reports^46^. Together, both datasets were used as our database as we understand that data reuse contributes to two of the four R’s of animal research (reduce and responsibility)^47,48^.

### Datasets

Weaned pigs dataset^18^ comprised behavioral observations of pigs in pre- and post-castration timepoints. There were 45 Landrace, Large White, Duroc and Hampshire male pigs randomly selected from the university commercial production. The animals were aged 38 + 3 days and weighed 11.06 + 2.28 kg, and were housed in iron pens (2.40 × 1.50 × 1.50 m of length x width x height) located side by side separated by bars in groups of five pigs. Before the surgery, pigs were submitted to bilateral local anesthesia with 0.5 mL of 1% lidocaine without vasoconstrictor (Xylestesin®, Cristália, Itapira, São Paulo, Brazil) injected subcutaneously at each incision line, parallel to the scrotum shaft, followed by 1 mL injected intratesticularly at each testicle, and the surgery was performed after five minutes. Surgical castration was always performed by the same trained surgeon. Details about surgical procedures and housing conditions were described in the previous study^18^. The pigs were recorded from 24 to 16 h before surgery (pain-free condition), 3.5 to 4 h after surgery (pain condition), and other timepoints from which observations were not used in this study. In each timepoint, animals were evaluated for at least four minutes. All video recordings were assessed by three observers according to UPAPS. They were referred to as Gold Standard, Observer 1 and Observer 2 in the original paper^15^. In the original study, all observers assessed all videos (phase 1) and repeated all video assessments after an interval (phase 2) due to psychometric validation steps, however, we used only the first phase of the assessment to merge two datasets, since in the second dataset (described below) was performed only one assessment phase.

Pre-weaned pigs dataset^19^ comprised behavioral observations of piglets in pre- and post-castration timepoints. There were 39 Yorkshire-Landrace x Duroc piglets enrolled in the study. The animals were aged five days and weighed 1.62 ± 0.23 kg, housed with sows in individual farrowing crates (0.8 × 2.3 m of length x width) in fully slatted floors in a farrowing room with controlled environment conditions. General or local anesthesia was not administered, as it is standard practice in the United States and the procedure followed the standard operating procedure approved by the attending veterinarian. All male piglets at this facility underwent castration prior to weaning, therefore the castration procedure would have occurred regardless of the research. Surgical castration was always performed by the same trained surgeon. Details about surgical procedures and housing conditions were described in the previous study^19^. However, all piglets enrolled in Pre-weaned pigs dataset did receive intramuscular flunixin meglumine (2.2 mg/kg flunixin meglumine IM; Merck Animal Health, Millsboro, DE, US) one hour after surgery. The animals were recorded at 24 h before surgery (pain-free condition), 15 min after surgery (pain condition), and other timepoints from which observations were not used in this study. The animals were recorded and video clips of 4 min were obtained. Some piglets were asleep, so we only considered assessments of awake piglets (n = 14). All video recordings were assessed by two observers in a single assessment phase.

First, both datasets were split separately into (i) a train set comprising 70% of pigs (31 weaned and 10 pre-weaned) selected randomly, used for algorithm fitting, and (ii) a test set with 30% of reminiscent pigs (14 weaned and 4 pre-weaned), used for algorithm predicting. Following, train and test sets from weaned and pre-weaned pigs datasets were merged. Then, both train and test sets contained five observers, two perioperative timepoints, and two age groups, changing the number of pigs and consequently the number of observations (410 and 180, respectively 70 and 30%).

### Pain-altered behavior scale

In the UPAPS, six behavioral items regarding posture, interaction and interest in the surroundings, activity, appetite (for weaned pigs), attention to the affected area and miscellaneous behaviors are assessed. These behavioral items are descriptive and composed by four score levels: ‘0’, ‘1’, ‘2’ and ‘3’, according to the presence or absence of pain-related behaviors (Table 4). In the UPAPS validation for pre-weaned pigs, the nursing behavior would be analogous to the appetite in weaned pigs, but Nursing item was disregarded for pre-weaned piglets^19^. In order to merge the databases, the appetite and nursing items were disregarded. Then, the total sum of the five behavioral items scores (0–15) were considered to assess pain.

**Table 4:** Unesp-Botucatu Pig Composite Pain Scale system without appetite or nursing item^18,19^.

Item	Score	Score/criterion	Links to videos
Posture	0	Normal (any position, apparent comfort, relaxed muscles) or sleeping	https://youtu.be/QSosCD2SD4E
	1	Changes posture, with discomfort	https://youtu.be/SpaWsFCrPxE
	2	Changes posture, with discomfort, and protects the affected area	https://youtu.be/VjSlsRrG8yA
	3	Quiet, tense, and back arched	https://youtu.be/pm4hJ5163ao
Interaction and interest in the surroundings	0	Interacts with other animals; interested in the surroundings or sleeping	https://youtu.be/-880STgYq2I
	1	Only interacts if stimulated by other animals; interested in the surroundings	https://youtu.be/nXjOdwn3dyw
	2	Occasionally moves away from the other animals, but accepts approaches; shows little interest in the surroundings	https://youtu.be/2k2JDr5U6As
	3	Moves or runs away from other animals and does not allow approaches; disinterested in the surroundings	https://youtu.be/se70oYXcWFw
Activity	0	Moves normally or sleeping	https://youtu.be/cC75t7L5-YA
	1	Moves with less frequency	https://youtu.be/lQo9wq8LAn8
	2	Moves constantly, restless	https://youtu.be/YQRJjijLvpk
	3	Reluctant to move or does not move	https://youtu.be/Zyx0G3Wpt8o
Attention to the affected area		A. Elevates pelvic limb or alternates the support of the pelvic limb	https://youtu.be/UD99ftO7HE0
		B. Scratches or rubs the painful area	https://youtu.be/7idfFk1harE
		C. Moves and/or runs away and/or jumps after injury of the affected area	https://youtu.be/u-Pqubom278
		D. Sits with difficulty	https://youtu.be/ETNEOCVV4h0
	0	All the above behaviors are absent	
	1	Presence of one of the above behaviors	
	2	Presence of two of the above behaviors	
	3	Presence of three or all the above behaviors	
Miscellaneous behaviors		A. Wags tail continuously and intensely	https://youtu.be/pU5dGZFNRHc
		B. Bites the bars or objects	https://youtu.be/cF3dsq7gMtk
		C. The head is below the line of the spinal column	https://youtu.be/ZcIgngclRpI
		D. Presents difficulty in overcoming obstacles (example: another animal)	https://youtu.be/HlvdOI3lGuY
	0	All the above behaviors are absent	
	1	Presence of one of the above behaviors	
	2	Presence of two of the above behaviors	
	3	Presence of three or all the above behaviors	

### Statistical description

All statistical procedures were performed in R language, using RStudio integrated development environment^49^ (Version 4.2.2; RStudio, Inc., Boston, MA, USA). The functions and packages were presented in the format ‘package::function’. p-values were considered significant when p ≤ 0.05 in all tests. Figures were colored using a color palette distinguishable for common kinds of colorblindness (ggplot2::scale_colour_viridis_d).

#### Multilevel binomial logistic regression (LR)

Logistic Regression is a classification technique widely used for different purposes^50^. In this study, we used it to compute the respective probability of each observation (pain assessment) on being classified as pain or pain-free condition. A full algorithm (Full LR), containing all predictor variables was created, and used as reference for an automated algorithm selection (glmulti::glmulti) referred to as best subsets technique. This technique finds the best candidate algorithms with optimized information criteria. To select the best subset of predictors, we considered the Bayesian information criterion (BIC), which penalizes the predictor inclusion, and therefore it contributes to finding the better fitting with less predictor’s algorithms. An exhaustive search was used to find the exact solution. The best BIC algorithm is referred to as Refined LR.

Both Full LR and Refined LR followed the same procedures. Algorithms were created in the train set using stats::glm, using condition as response variable (0 = absence of pain, corresponding to M1; and 1 = presence of pain, corresponding to M2). The behavioral items from UPAPS were converted into dummy variables (0 = absence and 1 = presence of each behavior) (fastDummies::dummy_columns), and then used as predictor variables. After algorithm fitting, the event probability of occurring (Condition classification as 1) was computed for each observation in the test set (stats::predict). Wald statistics generated from the algorithms were used to rank behaviors, as proposed previously^34^.

#### Canonical discriminant analysis (CDA)

Canonical Discriminant Analysis is a variation of the linear discriminant analysis with the related Fisher’s linear discriminant method. It finds a linear combination of features that may be used as a classifier or dimensionality reduction before classification^51^. In this study, we adapted CDA to use a binomial response variable, rather than a multiclass variable, and performed it to compare its classification along with binomial multiple logistic regression (LR) and principal component analysis (PCA, described next). A Full CDA, with all five items as variables, and a Refined CDA, without Activity item, were performed. Activity was withdrawn because the best subsets technique for LR indicated the removal of all pain-altered behaviors related with Activity, so it was needed for a fair comparison. Both Full and Refined CDA followed the same procedures.

Canonical discriminant analysis was performed using Condition as grouping factor and UPAPS behavioral items scores as discriminators, using MASS::lda in the train set. Coefficients of linear discriminants were used to predict the probability of presence of pain (Condition = 1) in each observation of the test set. The discriminant coefficients were used as CDA weightings to obtain a new total score. For this purpose, each UPAPS item was multiplied by its respective CDA weighting (discriminant coefficient), resulting in a new score for each item. The new scores were added, resulting in a new total score for Full CDA and for Refined CDA. Discriminant coefficients generated from the algorithms were also used to rank behavioral items, as proposed previously^52^.

#### Principal component analysis (PCA)

Principal component analysis was used as an unsupervised comparison to supervised technique (logistic regression and canonical discriminant analysis). Principal Component Analysis is a dimensionality reduction technique that retains data variation that also might be used for testing the multiple association between variables^53^. It is performed by reducing the number of variables into principal components (PCs), where the data variation is maximal^40^. Similarly to CDA, a Full PCA and a Refined PCA without Activity, for the same reason, were performed and followed the same procedures described in this section.

The number of PCs retained was defined by Horn's parallel analysis using psych::fa.parallel on the train set. This method compares the factors scree of the observed data to a randomly generated one, of a data matrix of the same size as ours. The correlation matrix used Pearson correlation. The method was computed after 1,001 simulated analyses performed.

PCA was then performed (stats::princomp) on the train set. Eigean values were calculated using the standard deviation of the principal components. Loading values were obtained using stats::loadings. The loading values were used to mutate the original scores in the test set, resulting in a new total score based on PCA weightings. The loading values were used as PCA weightings to obtain a new total score. For this purpose, each UPAPS item was multiplied by its respective PCA weighting (loading value), resulting in a new score for each item. The new scores were added, resulting in a new total score for Full PCA and for Refined PCA.

Loading values generated from the algorithms were also used to rank behavioral items, as proposed previously^35^.

#### Predictive capacity

The area under the curve (AUC) from receiver operating characteristic (ROC) curve is a widely used technique to evaluate the performance of a binary classifier system as its discrimination threshold varies^54^. A ROC curve was generated using the Condition classes (pain and free-pain) as a predictor variable and each one of the six algorithms predicted in the test set, using pROC::ROC. It was also generated a ROC curve using UPAPS original scores and UPAPS scores without the Activity item. This function returns the AUC and its respective confidence interval. Furthermore, threshold, sensitivity, sensibility, and their respective 95% of confidence intervals were obtained for each ROC curve using pROC::ci.coords. Threshold was calculated using the Youden method. Both ROC and its coordinates were generated using 95% of confidence and bootstrapping stratification of 1001 replicates.

DeLong test was used to compare the AUCs generated from UPAPS and each algorithm. If more than one algorithm was detected as different from UPAPS, they were tested between them. DeLong test was performed using pROC::roc.test.

### Supplementary Information

Supplementary Information 1.

Supplementary Information 2.

Supplementary Tables.

## Data Availability

Weaned pigs dataset^18^ and Pre-weaned pigs dataset^19^ were already publicly available in the supplementary material of their respective publications. Also, merged datasets analyzed during this study and the R script were included in its supplementary information files.
