# Supplementary material for: Simplified assessment of castration-induced pain in pigs using lower complexity algorithms
Source: Sci Rep. 2023 Dec 1;13:21237. doi: 10.1038/s41598-023-48551-1 (PMC10692155; doi:10.1038/s41598-023-48551-1)
Supplement: Supplementary file 2 — Supplementary Information 2. [file 41598_2023_48551_MOESM2_ESM.docx]

#------------------------Data loading and pre-processing-------------------------

## Importing database

rm(list=ls()) # Cleaning directory

set.seed(306)

# Importing train and test sets

df_pigs_70 = read.csv2("70pigFullrf.csv",h=T,d=",",na.strings=".")

df_pigs_30 = read.csv2("30pigFullrf.csv",h=T,d=",",na.strings=".")

#Filtering datasets

df_pigs_70 = filter(df_pigs_70,

Phases == "Phase.1"

& Observers != 'Observer.3'

& Dataset != "Lopez Solano 2023")

df_pigs_30 = filter(df_pigs_30,

Phases == "Phase.1"

& Observers != 'Observer.3'

& Dataset != "Lopez Solano 2023")

#Dummy variables

df_pigs_70 = dummy_columns(df_pigs_70, select_columns= c(

'Posture',

'Interaction',

'Activity',

'Appetite',

'Attention',

'Lift.Pelvic.Limb',

'Scratching.Rubbing',

'Walk.Away.Run',

'Sit.With.Difficulty',

'Continuously.Balances',

'Bite.Grill',

'Head.Down',

'Difficulty.Overcoming'

),

remove_selected_columns=F,

remove_first_dummy=F)

df_pigs_30 = filter(df_pigs_30, Phases == 'Phase.1'& Observers !='Observer.3')

df_pigs_30=dummy_columns(df_pigs_30,select_columns=c(

'Posture',

'Interaction',

'Activity',

'Appetite',

'Attention',

'Lift.Pelvic.Limb',

'Scratching.Rubbing',

'Walk.Away.Run',

'Sit.With.Difficulty',

'Continuously.Balances',

'Bite.Grill',

'Head.Down',

'Difficulty.Overcoming'

),

remove_selected_columns=F,

remove_first_dummy=F)

#------------------------------Fitting models: LR-----------------------------------

#1- Full GLM

full_glm = glm(Condition~

+Posture_Posture.1

+Posture_Posture.2

+Posture_Posture.3

+Interaction_Interaction.1

+Interaction_Interaction.2

+Interaction_Interaction.3

+Activity_Activity.1

+Activity_Activity.2

+Activity_Activity.3

+Lift.Pelvic.Limb_Lift.Pelvic.Limb.1

+Scratching.Rubbing_Scratching.Rubbing.1

+Walk.Away.Run_Walk.Away.Run.1

+Sit.With.Difficulty_Sit.With.Difficulty.1

+Continuously.Balances_Continuously.Balances.1

+Bite.Grill_Bite.Grill.1

+Head.Down_Head.Down.1

+Difficulty.Overcoming_Difficulty.Overcoming.1

,family='binomial'

,df_pigs_70)

#write.csv2(summary(full_glm)$coefficients, "Table 1. Wald Statistics (Full model).csv")

#2- Best GLM (Criterion:AIC)

#model_selection = glmulti(Condition~

# +Posture_Posture.1

# +Posture_Posture.2

# +Posture_Posture.3

# +Interaction_Interaction.1

# +Interaction_Interaction.2

# +Interaction_Interaction.3

# +Activity_Activity.1

# +Activity_Activity.2

# +Activity_Activity.3

# +Lift.Pelvic.Limb_Lift.Pelvic.Limb.1

# +Scratching.Rubbing_Scratching.Rubbing.1

# +Walk.Away.Run_Walk.Away.Run.1

# +Sit.With.Difficulty_Sit.With.Difficulty.1

# +Continuously.Balances_Continuously.Balances.1

# +Bite.Grill_Bite.Grill.1

# +Head.Down_Head.Down.1

# +Difficulty.Overcoming_Difficulty.Overcoming.1,

# method="g",

# fitfunction = "glm",

# data=df_pigs_70,

# family="binomial",

# level=1,

# crit = "bic")

#best_AIC_model = glm(Condition~1+Posture_Posture.1

# +Posture_Posture.2

# +Interaction_Interaction.1

# +Interaction_Interaction.2

# +Interaction_Interaction.3

# +Scratching.Rubbing_Scratching.Rubbing.1

# +Continuously.Balances_Continuously.Balances.1

# +Bite.Grill_Bite.Grill.1

# +Head.Down_Head.Down.1,

# df_pigs_70, family="binomial")

best_BIC_model = glm(Condition~1+Posture_Posture.1

+Posture_Posture.2

+Interaction_Interaction.2

+Interaction_Interaction.3

+Continuously.Balances_Continuously.Balances.1

+Head.Down_Head.Down.1,

df_pigs_70, family="binomial")

#write.csv2(summary(best_BIC_model)$coefficients, "Table 2. Wald Statistics (Best BIC GLM).csv")

#4- Stepwise selected model (Criterion:AIC) (not gonna use for now)

#stepwise = step(glm(Condition~

# +Posture_Posture.1

# +Posture_Posture.2

# +Posture_Posture.3

# +Interaction_Interaction.1

# +Interaction_Interaction.2

# +Interaction_Interaction.3

# +Activity_Activity.1

# +Activity_Activity.2

# +Activity_Activity.3

# +Lift.Pelvic.Limb_Lift.Pelvic.Limb.1

# +Scratching.Rubbing_Scratching.Rubbing.1

# +Walk.Away.Run_Walk.Away.Run.1

# +Sit.With.Difficulty_Sit.With.Difficulty.1

# +Continuously.Balances_Continuously.Balances.1

# +Bite.Grill_Bite.Grill.1

# +Head.Down_Head.Down.1

# +Difficulty.Overcoming_Difficulty.Overcoming.1

# ,family='binomial'

# ,df_pigs_70),direction="both",steps=1001,k=qchisq(p=.05,df=1,lower.tail=F))

#step_selected_model = glm(Condition ~ Posture_Posture.1

# + Posture_Posture.2

# + Posture_Posture.3

# + Interaction_Interaction.1

# + Interaction_Interaction.2

# + Walk.Away.Run_Walk.Away.Run.1

# + Scratching.Rubbing_Scratching.Rubbing.1

# + Continuously.Balances_Continuously.Balances.1

# + Head.Down_Head.Down.1

# ,family="binomial"

# ,df_pigs_70)

#------------------------------Fitting models: PCA------------------------

#-----Full PCA (With Activity)

#psych::fa.parallel(df_pigs_70[,c(9:13)],fa="pc",n.iter=1001,cor='cor',show.legend=F)

# Running PCA

pca=princomp(df_pigs_70[,c(9:13)], cor=T)

# Extracting PCA metrics

#eig=(pca$sdev)^2

#variance=eig*100/sum(eig)

#cumvar=cumsum(variance)

#eigenvalue=data.frame(eig=eig,variance=variance,

# cumvariance=cumvar);eigenvalue

load = loadings(pca)

# Calculating new score

df_pigs_30 = mutate(df_pigs_30,

Posture_PCA = Posture.num*load[1],

Interaction_PCA = Interaction.num*load[2],

Activity_PCA = Activity.num*load[3],

Attention_PCA = Attention.num*load[4],

Miscellaneous_PCA = Miscellaneous.num*load[5],

PCA_sum = Posture_PCA+Interaction_PCA+Attention_PCA+Miscellaneous_PCA)

#-----Refined PCA (Without Activity)

#psych::fa.parallel(df_pigs_70[,c(9:10, 12:13)],fa="pc",n.iter=1001,cor='cor',show.legend=F)

# Running PCA

pca1=princomp(df_pigs_70[,c(9:10, 12:13)], cor=T)

# Extracting PCA metrics

#eig=(pca$sdev)^2

#variance=eig*100/sum(eig)

#cumvar=cumsum(variance)

#eigenvalue=data.frame(eig=eig,variance=variance,

# cumvariance=cumvar);eigenvalue

load1 = loadings(pca1)

# Calculating new score

df_pigs_30 = mutate(df_pigs_30,

Posture_PCA1 = Posture.num*load1[1],

Interaction_PCA1 = Interaction.num*load1[2],

Attention_PCA1 = Attention.num*load1[3],

Miscellaneous_PCA1 = Miscellaneous.num*load1[4],

PCA_sum1 = Posture_PCA1+Interaction_PCA1+Attention_PCA1+Miscellaneous_PCA1)

#------------------------------Fitting models: CDA-----------------------------

# Full CDA (with Activity)

model_cda = lda(Condition ~ Posture.num

+Interaction.num

+Activity.num

+Attention.num

+Miscellaneous.num,

data = df_pigs_70)

df_pigs_30 = mutate(df_pigs_30,

Posture_CDA = Posture.num*model_cda$scaling[1],

Interaction_CDA = Interaction.num*model_cda$scaling[2],

Activity_CDA = Activity.num*model_cda$scaling[3],

Attention_CDA = Attention.num*model_cda$scaling[4],

Miscellaneous_CDA = Miscellaneous.num*model_cda$scaling[5],

CDA_sum = Posture_CDA+Interaction_CDA+Attention_CDA+Miscellaneous_CDA)

#write.csv2(model_cda$scaling, "Table 5. Normalized discriminant functions (CDA).csv")

# Refined CDA (withou Activity)

model_cda1 = lda(Condition ~ Posture.num

+Interaction.num

+Attention.num

+Miscellaneous.num,

data = df_pigs_70)

df_pigs_30 = mutate(df_pigs_30,

Posture_CDA1 = Posture.num*model_cda1$scaling[1],

Interaction_CDA1 = Interaction.num*model_cda1$scaling[2],

Attention_CDA1 = Attention.num*model_cda1$scaling[3],

Miscellaneous_CDA1 = Miscellaneous.num*model_cda1$scaling[4],

CDA_sum1 = Posture_CDA1+Interaction_CDA1+Attention_CDA1+Miscellaneous_CDA1)

#write.csv2(model_cda2$scaling, "Table 6. Normalized discriminant functions (CDA refined).csv")

#-----------------------------Predicting models---------------------------------

df_pigs_30$prob1 = predict(full_glm, newdata=df_pigs_30, type='response')

df_pigs_30$prob2 = predict(best_BIC_model, newdata=df_pigs_30, type='response')

df_pigs_30$prob_cda <- predict(model_cda, newdata = df_pigs_30, type="response")$posterior[, "1"]

df_pigs_30$prob_cda1 <- predict(model_cda1, newdata = df_pigs_30, type="response")$posterior[, "1"]

roc_UPAPS = roc(Condition~UPAPS.new, df_pigs_30, plot=F,

algorithm=2, smooth=F, boot.n=1001, boot.stratified=T, ci.auc=T, auc=T)

roc_UPAPS_cicoords = ci.coords(roc_UPAPS,x="best",

input=c("threshold", "specificity", "sensitivity"),

ret=c("threshold", "specificity", "sensitivity"),

best.method="youden",

best.policy = "random",

conf.level=0.95, boot.n=1001,

boot.stratified=TRUE)

roc_UPAPS2 = roc(Condition~UPAPS.wo.Activity, df_pigs_30, plot=F,

algorithm=2, smooth=F, boot.n=1001, boot.stratified=T, ci.auc=T, auc=T)

roc_UPAPS2_cicoords = ci.coords(roc_UPAPS2,x="best",

input=c("threshold", "specificity", "sensitivity"),

ret=c("threshold", "specificity", "sensitivity"),

best.method="youden",

best.policy = "random",

conf.level=0.95, boot.n=1001,

boot.stratified=TRUE)

roc_fullglm = roc(Condition~prob1, df_pigs_30, plot=F,

algorithm=2, smooth=F, boot.n=1001, boot.stratified=T, ci.auc=T, auc=T)

roc_fullglm_cicoords = ci.coords(roc_fullglm,x="best",

input=c("threshold", "specificity", "sensitivity"),

ret=c("threshold", "specificity", "sensitivity"),

best.method="youden",

best.policy = "random",

conf.level=0.95, boot.n=1001,

boot.stratified=TRUE)

roc_bestBIC = roc(Condition~prob2, df_pigs_30, plot=F,

algorithm=2, smooth=F, boot.n=1001, boot.stratified=T, ci.auc=T, auc=T)

roc_bestBIC_cicoords = ci.coords(roc_bestBIC,x="best",

input=c("threshold", "specificity", "sensitivity"),

ret=c("threshold", "specificity", "sensitivity"),

best.method="youden",

best.policy = "random",

conf.level=0.95, boot.n=1001,

boot.stratified=TRUE)

roc_pca = roc(Condition~PCA_sum,df_pigs_30,plot=F,

algorithm=2,smooth=F,boot.n=1001,boot.stratified=T,ci.auc=T,auc=T)

roc_pca_ci_coords = ci.coords(roc_pca,x="best",

input=c("threshold", "specificity", "sensitivity"),

ret=c("threshold", "specificity", "sensitivity"),

best.method="youden",

best.policy = "random",

conf.level=0.95, boot.n=1001,

boot.stratified=TRUE)

roc_pca1 = roc(Condition~PCA_sum1,df_pigs_30,plot=F,

algorithm=2,smooth=F,boot.n=1001,boot.stratified=T,ci.auc=T,auc=T)

roc_pca_ci_coords1 = ci.coords(roc_pca1,x="best",

input=c("threshold", "specificity", "sensitivity"),

ret=c("threshold", "specificity", "sensitivity"),

best.method="youden",

best.policy = "random",

conf.level=0.95, boot.n=1001,

boot.stratified=TRUE)

roc_cda = roc(Condition~CDA_sum, df_pigs_30, plot=F,

algorithm=2, smooth=F, boot.n=1001, boot.stratified=T, ci.auc=T, auc=T)

roc_cda_cicoords = ci.coords(roc_cda,x="best",

input=c("threshold", "specificity", "sensitivity"),

ret=c("threshold", "specificity", "sensitivity"),

best.method="youden",

best.policy = "random",

conf.level=0.95, boot.n=1001,

boot.stratified=TRUE)

roc_cda1 = roc(Condition~CDA_sum1, df_pigs_30, plot=F,

algorithm=2, smooth=F, boot.n=1001, boot.stratified=T, ci.auc=T, auc=T)

roc_cda1_cicoords = ci.coords(roc_cda1,x="best",

input=c("threshold", "specificity", "sensitivity"),

ret=c("threshold", "specificity", "sensitivity"),

best.method="youden",

best.policy = "random",

conf.level=0.95, boot.n=1001,

boot.stratified=TRUE)

#-------------------------------DeLong test-------------------------------

test1 = roc.test(roc_UPAPS, roc_fullglm); test1$p.value

test2 = roc.test(roc_UPAPS, roc_bestBIC); test2$p.value

test3 = roc.test(roc_UPAPS, roc_pca); test3$p.value

test4 = roc.test(roc_UPAPS, roc_pca1); test4$p.value

test5 = roc.test(roc_UPAPS, roc_cda); test5$p.value

test6 = roc.test(roc_UPAPS, roc_cda1); test6$p.value

test7 = roc.test(roc_UPAPS, roc_UPAPS2); test7$p.value

#-------------------------------Computing results--------------------------------

model_names = c("UPAPS", "LR", "Refined LR", "PCA", "Refined PCA", "CDA", "Refined CDA")

models_AUC = c(roc_UPAPS$auc[1],

roc_fullglm$auc[1],

roc_bestBIC$auc[1],

roc_pca$auc[1],

roc_pca1$auc[1],

roc_cda$auc[1],

roc_cda1$auc[1]); models_AUC = round(models_AUC, digits=4)

models_AUC_lower = c(ci.auc(roc_UPAPS)[1],

ci.auc(roc_fullglm)[1],

ci.auc(roc_bestBIC)[1],

ci.auc(roc_pca)[1],

ci.auc(roc_pca1)[1],

ci.auc(roc_cda)[1],

ci.auc(roc_cda1)[1]); models_AUC_lower = round(models_AUC_lower, digits=4)

models_AUC_higher = c(ci.auc(roc_UPAPS)[3],

ci.auc(roc_fullglm)[3],

ci.auc(roc_bestBIC)[3],

ci.auc(roc_pca)[3],

ci.auc(roc_pca1)[3],

ci.auc(roc_cda)[3],

ci.auc(roc_cda1)[3]); models_AUC_higher = round(models_AUC_higher, digits=4)

pvalue_UPAPS = c(NA,

test1$p.value,

test2$p.value,

test3$p.value,

test4$p.value,

test5$p.value,

test6$p.value); pvalue_UPAPS = round(pvalue_UPAPS, digits=3)

threshold_lower = c(roc_UPAPS_cicoords$threshold[1],

roc_fullglm_cicoords$threshold[1],

roc_bestBIC_cicoords$threshold[1],

roc_pca_ci_coords$threshold[1],

roc_pca_ci_coords1$threshold[1],

roc_cda_cicoords$threshold[1],

roc_cda1_cicoords$threshold[1]); threshold_lower = round(threshold_lower, 4)

threshold_median = c(roc_UPAPS_cicoords$threshold[2],

roc_fullglm_cicoords$threshold[2],

roc_bestBIC_cicoords$threshold[2],

roc_pca_ci_coords$threshold[2],

roc_pca_ci_coords1$threshold[2],

roc_cda_cicoords$threshold[2],

roc_cda1_cicoords$threshold[2]); threshold_median = round(threshold_median, 4)

threshold_higher = c(roc_UPAPS_cicoords$threshold[3],

roc_fullglm_cicoords$threshold[3],

roc_bestBIC_cicoords$threshold[3],

roc_pca_ci_coords$threshold[3],

roc_pca_ci_coords1$threshold[3],

roc_cda_cicoords$threshold[3],

roc_cda1_cicoords$threshold[3]); threshold_higher = round(threshold_higher, 4)

sensitivity_lower = c(roc_UPAPS_cicoords$sensitivity[1],

roc_fullglm_cicoords$sensitivity[1],

roc_bestBIC_cicoords$sensitivity[1],

roc_pca_ci_coords$sensitivity[1],

roc_pca_ci_coords1$sensitivity[1],

roc_cda_cicoords$sensitivity[1],

roc_cda1_cicoords$sensitivity[1]); sensitivity_lower = round(sensitivity_lower, 4)

sensitivity_median = c(roc_UPAPS_cicoords$sensitivity[2],

roc_fullglm_cicoords$sensitivity[2],

roc_bestBIC_cicoords$sensitivity[2],

roc_pca_ci_coords$sensitivity[2],

roc_pca_ci_coords1$sensitivity[2],

roc_cda_cicoords$sensitivity[2],

roc_cda1_cicoords$sensitivity[2]); sensitivity_median = round(sensitivity_median, 4)

sensitivity_higher = c(roc_UPAPS_cicoords$sensitivity[3],

roc_fullglm_cicoords$sensitivity[3],

roc_bestBIC_cicoords$sensitivity[3],

roc_pca_ci_coords$sensitivity[3],

roc_pca_ci_coords1$sensitivity[3],

roc_cda_cicoords$sensitivity[3],

roc_cda1_cicoords$sensitivity[3]); sensitivity_higher = round(sensitivity_higher, 4)

specificity_lower = c(roc_UPAPS_cicoords$specificity[1],

roc_fullglm_cicoords$specificity[1],

roc_bestBIC_cicoords$specificity[1],

roc_pca_ci_coords$specificity[1],

roc_pca_ci_coords1$specificity[1],

roc_cda_cicoords$specificity[1],

roc_cda1_cicoords$specificity[1]); specificity_lower = round(specificity_lower, 4)

specificity_median = c(roc_UPAPS_cicoords$specificity[2],

roc_fullglm_cicoords$specificity[2],

roc_bestBIC_cicoords$specificity[2],

roc_pca_ci_coords$specificity[2],

roc_pca_ci_coords1$specificity[2],

roc_cda_cicoords$specificity[2],

roc_cda1_cicoords$specificity[2]); specificity_median = round(specificity_median, 4)

specificity_higher = c(roc_UPAPS_cicoords$specificity[3],

roc_fullglm_cicoords$specificity[3],

roc_bestBIC_cicoords$specificity[3],

roc_pca_ci_coords$specificity[3],

roc_pca_ci_coords1$specificity[3],

roc_cda_cicoords$specificity[3],

roc_cda1_cicoords$specificity[3]); specificity_higher = round(specificity_higher, 4)

results = data.frame("Model" = model_names,

"AUC95" = models_AUC,

"CILower95" = models_AUC_lower,

"CIHigher95" = models_AUC_higher,

"UPAPS.pvalue" = pvalue_UPAPS,

"Threshold" = threshold_median,

"Threshold.min" = threshold_lower,

"Threshold.max" = threshold_higher,

"Sensitivity" = sensitivity_median,

"Sensitivity.min" = sensitivity_lower,

"Sensitivity.max" = sensitivity_higher,

"Specificity" = specificity_median,

"Specificity.min" = specificity_lower,

"Specificity.max" = specificity_higher)

#write.csv2(results, "resultsROC.csv")
