# Supplementary material for: Simplified assessment of castration-induced pain in pigs using lower complexity algorithms
Source: Sci Rep. 2023 Dec 1;13:21237. doi: 10.1038/s41598-023-48551-1 (PMC10692155; doi:10.1038/s41598-023-48551-1)
Supplement: Supplementary file 3 — Supplementary Tables. [file 41598_2023_48551_MOESM3_ESM.docx]

**Supplementary material**

**Supplementary Table S1.** Full principal component analysis eigenvalues, variance and cumulative variance of principal components.

| **Components** | **Eigenvalues** | **Variance (%)** | **Cumulative variance (%)** |
| --- | --- | --- | --- |
| PC1 | 3.62 | 72.45 | 72.45 |
| PC2 | 0.51 | 10.23 | 82.68 |
| PC3 | 0.36 | 7.11 | 89.79 |
| PC4 | 0.31 | 6.20 | 95.99 |
| PC5 | 0.20 | 4.00 | 100 |

**Supplementary Table S2.** Refined principal component analysis eigenvalues, variance and cumulative variance of principal components.

| **Components** | **Eigenvalues** | **Variance (%)** | **Cumulative variance (%)** |
| --- | --- | --- | --- |
| PC1 | 3.04 | 76.16 | 76.16 |
| PC2 | 0.40 | 9.97 | 86.13 |
| PC3 | 0.35 | 8.63 | 94.77 |
| PC4 | 0.21 | 5.23 | 100 |
